# Dental Association of Canada West

**Published:** 1867-01

**Authors:** J. S. Scott

**Affiliations:** Cobourg, C. W.


*Secretary.*

---

### DENTAL ASSOCIATION OF CANADA WEST.

BY J. S. SCOTT, COBOURG, C. W.

A convention of Dentists was held in Toronto, January 3d, 1867. Present: B. W. Day, M. D., of Kingston; Drs. C. S. Chittenden, of Hamilton; F. G. Callender, of Cobourg; J. O'Donnell, of Peterboro'; A. D. Lalonde, of Brockville, M. E. Snider, of Toronto; D. A. Hogart, of Hamilton; J. S. Scott of Cobourg. B. W. Day, M. D., appointed Chairman, and J. S. Scott, Secretary.

The Secretary read the circular calling the meeting. Letters were received from Drs. G. V. N. Relyea, of Belleville —S. T. Clements, of Napanee—and from most of the established Dentists from all sections of Canada West not in attendance, approving of the object of the convention, and stating their inability to attend until next meeting.

The chairman then stated that the object of the convention was to organize a Dental Association, and to take steps to procure the passing of a law, requiring Dentists to undergo an examination.

Drs. F. G. Callender, J. O'Donnell, and H. T. Wood, expressed themselves in favor of organizing an Association, and of securing an act of incorporation. Dr. S. C. Chittenden said he was pleased to see so large an attendance. That unless we organized an Association at this meeting, he feared we could not get so many together again in a long time. He did not look upon a protective law of so much consequence as the great advantages to be derived from associating together and comparing ideas of practice. He would not oppose Incorporation, but desired first an Association.

Dr. Lalonde said he was in favor of procuring a law requiring Dentists to have license, and of preventing those unqualified from practising. He was surrounded by quack Dentists, who were travelling the country over without an office or even a Post-office address, imposing upon unsuspecting people, and injuring qualified Dentists, who were obliged to keep up established offices.

Dr. J. S. Scott said it was time the people had some means of knowing who were qualified to practice as Dentists. He was in favor of securing an Act requiring Dentists to pass an examination. That the first action of this meeting should be to organize a society. He would therefore move, seconded by Dr. C. S. Chittenden, "That we proceed to the organization of a Dental Association for Canada West." Carried.

On motion—Drs. F. G. Callender, C. S. Chittenden, H. T. Wood, J. O'Donnell and the chairman were appointed a Committee to draft a Constitution. The committee reported a draft of Constitution which was adopted: requiring that candidates for membership, in addition to professional knowledge, shall have practised successfully for five years in one place, in an established office. Students who are articled to regular practitioners for not less than two years may become *incipient members*, on ballot, by being recommended by two members of the Association.

The following officers were then elected :—

B. W. Day, M. D., President; Dr. C. S. Chittenden, 1st Vice President; Dr. H. T. Wood, 2d Vice President; Dr. F. G. Callender, Treasurer; Dr. J. S. Scott, Recording Secretary; Dr. J. O'Donnell, Corresponding Secretary; Dr. D. A. Bogart, Librarian. Committee on By-Laws—Drs. F. G. Callender, D. A. Bogart, M. E. Snider, and A. D. Lalonde.

Moved by Dr. D. A. Bogart, seconded by Dr. G. Callender:

That the following committee to draft a Bill of Incorporation, to be submitted to Parliament at its next session: The Committee to consist of the President, Vice Presidents, and Secretaries. Carried.

Moved by Dr. C. S. Chittenden, seconded by Dr. D. A. Bogart—

That Dr. G. V. N. Relyea of Belleville, be elected a member of this Association, he being unavoidably prevented from attending this meeting. Carried unanimously.

Moved by Dr. J. S. Scott, seconded by Dr. D. A. Bogart—

That the following members be requested to read papers at the next meeting as follows: Dr. F. G. Callender, upon Operative Dentistry; Dr. G. V. N. Relyea, upon Nitrous Oxide Gas; Dr. J. O'Donnell, upon Diseases of the Antrum; Dr. C. S. Chittenden to select his subject. Carried.

Dr. C. S. Chittenden said this Association having adopted a high standard of qualification, it was desirable the public should know, who had become members. He would therefore move, seconded by Dr. T. H. Wood—

That the members be requested to insert in their cards the following—"Member of the Dental Association of Canada West." Carried.

The semi-annual session will be held in Cobourg, on the first Tuesday in July next, to commence at 6 o'clock P. M. The next annual session at Hamilton, on the third Tuesday in January next, to commence at 7 o'clock, P. M.

